# Editorial: Protein crops: food and feed for the future, volume II

**DOI:** 10.3389/fpls.2023.1271749

**Published:** 2023-08-28

**Authors:** Antonio M. De Ron, Francesca Sparvoli, Didier Bazile, A. Paula Rodiño

**Affiliations:** ^1^ Biology of Agrosystems, Misión Biológica de Galicia, Spanish National Research Council (MBG-CSIC), Pontevedra, Spain; ^2^ Institute of Agricultural Biology and Biotechnology, Consiglio Nazionale delle Ricerche (IABB-CNR), Milan, Italy; ^3^ CIRAD, UMR SENS, Montpellier, France; ^4^ UMR SENS, CIRAD, IRD, Univ Paul Valery Montpellier 3, Univ Montpellier, Montpellier, France

**Keywords:** protein crops, grain legumes, pseudocereals, soybean, pea, chickpea, bean

Grain legumes and pseudocereals, such as quinoa (*Chenopodium quinoa* Wild.), are the main crops cultivated to satisfy the growing world demand for plant protein for food and feed (De Ron, 2015; De Ron et al., 2017; Jiménez and Clemente, 2017; Kole, 2019).

Soybean [*Glycine max* (L.) Merr.] is the most cultivated legume in the world (391 × 10^6^ Mg in 2022/2023, World Agricultural Production, 2023). Ramlal et al. reviewed the harnessing heterosis and male sterility in soybean as it is of prime importance because it ensures the success of any breeding by enhancing the outcomes and results of this protein crop. In many crops, heterosis is mainly limited to the improvement of grain yield and is considered the basis for the development of more productive varieties. Male sterility is extensively used for the production of seeds and the improvement of crops together with traditional breeding programs and molecular technology.

In the mechanical harvest of grain legumes, the architecture of the plants is relevant. Kuzbakova et al. evaluated the height from the soil to the first pod (HFP) in several grain legumes (soybean, common bean, chickpea, pea, faba bean, lentil, cowpea, bitter vetch, cluster bean, and fenugreek), which is relevant for mechanical harvesting. They present a schematic representation summarising the model of HFP, and how and which other traits, factors, and genes interact and influence HFP. To minimise the loss of pods, the HFP must be higher than that of the blades of most combine harvesters. Here, the genetic control, morphology, and variability of HFP in legumes were revised, and there was an attempt to unravel the diverse terminology for this trait in the reviewed literature. Only a few publications have described a QTL analysis in which candidate genes for HFP with confirmed gene expression have been mapped. The authors found no information about simple and efficient markers associated with HFP, which could be used for marker-assisted selection for this trait.

Pea (*Pisum sativum* L.) is a protein crop cultivated worldwide, both for human food and animal feed. Some varieties of field pea improved through breeding for yield and disease resistance were studied by Zhao et al. Four yield trials representing the advancement stages of the breeding program for the breeding lines were assessed through grain yield, aerial high-throughput phenotyping (NDVI), and bacterial blight disease scores. Low-to-moderate broad-sense heritability (0.31–0.71) and narrow-sense heritability (0.13–0.71) were observed, as the estimated additive and non-additive genetic components for the three traits varied with the different models fitted. The study suggested that NDVI is a more useful trait for predicting grain yield, with high accuracy in the field pea breeding program, especially in diseased trials, through its incorporation into multivariate models. Wrinkled vining pea genotypes comprising historical varieties and breeding lines were studied by Alemu et al. to detect molecular markers associated with agronomic traits relevant to pea production. Marker-trait associations (MTAs) were investigated using 6,902 quality single nucleotide polymorphism (SNP) markers generated from the diversity arrays technology sequencing (DArTseq) and genotyping-by-sequencing (GBS) sequencing methods. They reported several novel MTAs for different crucial traits with agronomic importance in wrinkled vining pea production for the first time, and these candidate markers could be easily validated and integrated into the active breeding programs for marker-assisted selection.

Traoré et al. evaluated chickpea (*Cicer arietinum* L.) breeding lines grown under field conditions to reveal their agronomic performance and nutritional quality value. Only four genotypes of the 282 included in the study combined both good agronomic performance and high nutritional quality. Correlation analysis indicated that the studied traits were significantly intercorrelated, with a negative correlation between protein content and Zn concentration. Positive correlations were observed between grain filling time and the micronutrients Zn, Cu, and Mn and macroelements K and Mg. A low positive correlation was also recorded between protein and Fe concentrations. Positive correlations observed between main agronomic and nutritional quality traits could help in the simultaneous genetic improvement of these traits.

Common bean (*Phaseolus vulgaris* L.) is the main grain legume cultivated in the world for direct human consumption due to its nutritional properties. The dietary fibre (DF) content in dry bean was studied by Brick et al. with the objective of measuring the content of soluble and insoluble fibre and oligosaccharides in a diverse panel of 275 bean varieties. Insoluble dietary fibre constituted the highest portion of total DF (54.0%), followed by soluble dietary fibre (29.1%) and oligosaccharides (16.8%). Mean total DF and all components did not differ among genotypes grown in two field environments. These results indicate that value could be added to dry bean by cultivar-specific food labelling for protein and components of dietary fibre. Cominelli et al. reviewed the antinutritional factors, nutritional improvement, and future food use of common bean, including new common bean genotypes with reduced antinutritional compounds discovered through the exploitation of the natural genetic variability of common bean and the application of induced mutagenesis. The purpose of this Perspective paper is to first highlight the current advances in mutant bean characterisation and explore further advantages and disadvantages of these bean mutants and their potential use in innovative foods. A view is provided on future research directions to specifically explore further advantages and disadvantages of these bean mutants, their potential use in innovative foods, and their utility as a valuable genetic reservoir of combinations to assess the true functional role of specific seed bioactive components directly in the food matrix.

In the context of climate change, quinoa represents a potential alternative crop for increasing crop diversity, agricultural productivity, and farmers’ income in dryland areas. Taaime et al. studied the appropriate crop management practices with a limited water supply, which are still poorly documented in quinoa production. To determine the adequate levels of nutrient requirements and their effect on quinoa growth and productivity, a field experiment was conducted during two growing seasons; the treatments studied were combinations of several N, P, and K rates. The field results provide recommendations for quinoa cultivation in dryland areas.

As a general conclusion, it can be considered that the papers published in this Research Topic review important aspects of worldwide protein crops (soybean, common bean, chickpea, pea, faba bean, lentil, cowpea, bitter vetch, cluster bean, fenugreek, and quinoa). Advances in relevant areas, such as the genetic aspects of breeding, grain harvesting, crop diseases, nutritional qualities, and adaptation to environmental conditions, are compiled.

## Author contributions

AMR: Writing – original draft, Writing – review & editing. FS: Writing – original draft, Writing – review & editing. DB: Writing – original draft, Writing – review & editing. APR: Writing – original draft, Writing – review & editing.
